# Hospital Safety Culture in Taiwan: A Nationwide Survey Using Chinese Version Safety Attitude Questionnaire

**DOI:** 10.1186/1472-6963-10-234

**Published:** 2010-08-10

**Authors:** Wui-Chiang Lee, Hwei-Ying Wung, Hsun-Hsiang Liao, Chien-Ming Lo, Fei-Ling Chang, Pa-Chun Wang, Angela Fan, Hsin-Hsin Chen, Han-Chuan Yang, Sheng-Mou Hou

**Affiliations:** 1 Department of Medical Affairs and Planning, Taipei Veterans General Hospital, and Institute of Hospital and Health Care Administration, National Yang-Ming University School of Medicine, Taipei, Taiwan; 2 Taiwan Joint Commission on Hospital Accreditation, Taipei, Taiwan; 3 Department of Quality Improvement, Taiwan Joint Commission on Hospital Accreditation, Taipei, Taiwan; 4 Fu Jen Catholic University School of Medicine, Taipei County, Taiwan, Quality Management Center, Cathay General Hospital, Taipei, Taiwan; 5 National Yang-Ming University School of Medicine, Taipei, Taiwan; 6 National Health Research Institutes, Miaoli, Taiwan; 7 Department of Healthcare Administration, Asia University, Taichung, Taiwan; 8 Shin Kong Wu Ho-Su Memorial Hospital, Taipei, Taiwan

## Abstract

**Background:**

Safety activities have been initiated at many hospitals in Taiwan, but little is known about the safety culture at these hospitals. The aims of this study were to verify a safety culture survey instrument in Chinese and to assess hospital safety culture in Taiwan.

**Methods:**

The Taiwan Patient Safety Culture Survey was conducted in 2008, using the adapted Safety Attitude Questionnaire in Chinese (SAQ-C). Hospitals and their healthcare workers participated in the survey on a voluntary basis. The psychometric properties of the five SAQ-C dimensions were examined, including teamwork climate, safety climate, job satisfaction, perception of management, and working conditions. Additional safety measures were asked to assess healthcare workers' attitudes toward their collaboration with nurses, physicians, and pharmacists, respectively, and perceptions of hospitals' encouragement of safety reporting, safety training, and delivery delays due to communication breakdowns in clinical areas. The associations between the respondents' attitudes to each SAQ-C dimension and safety measures were analyzed by generalized estimating equations, adjusting for the clustering effects at hospital levels.

**Results:**

A total of 45,242 valid questionnaires were returned from 200 hospitals with a mean response rate of 69.4%. The Cronbach's alpha was 0.792 for teamwork climate, 0.816 for safety climate, 0.912 for job satisfaction, 0.874 for perception of management, and 0.785 for working conditions. Confirmatory factor analyses demonstrated a good model fit for each dimension and the entire construct. The percentage of hospital healthcare workers holding positive attitude was 48.9% for teamwork climate, 45.2% for perception of management, 42.1% for job satisfaction, 37.2% for safety climate, and 31.8% for working conditions. There were wide variations in the range of SAQ-C scores in each dimension among hospitals. Compared to those without positive attitudes, healthcare workers with positive attitudes to each SAQ dimension were more likely to perceive good collaboration with coworkers, and their hospitals were more likely to encourage safety reporting and to prioritize safety training programs (Wald chi-square test, p < 0.001 for all).

**Conclusions:**

Analytical results verified the psychometric properties of the SAQ-C at Taiwanese hospitals. The safety culture at most hospitals has not fully developed and there is considerable room for improvement.

## Background

Safety experts believe that patient safety begins with the enforcement of system safety of healthcare organizations [1-3]. Vincent *et al. *suggested that an organization's safety culture is a fundamental factor that influences system safety [2]. Safety culture is typically defined as "the shared attitudes, beliefs, values and assumptions that underlie how people perceive and act upon safety issues within their organizations" [4]. The term "safety climate" generally refers to the outward expression or measurable components of "safety culture" such as management behaviors, safety systems, and employee perceptions of safety [5]. Although the exact meanings of 'safety culture" and "safety climate" are different, these two terms have been used interchangeably in daily work and in previous studies. The focus of this study was on hospital's safety climate, but we adopted the term "safety culture" because it is more commonly used in Taiwan and in previous studies than "safety climate."

Organizational safety culture can be assessed using psychometric questionnaires that measure collective attitudes of personnel within the organization [5,6]. High-risk businesses, such as those in the aviation industry, have regularly evaluated employees' safety attitudes and their organizational safety culture for over 20 years [7,8]. Healthcare organizations are now becoming aware of the importance of measuring and transforming organizational culture to ensure patient safety [6,9,10]. The need for assessment tools to evaluate the cultural aspects of patient safety efforts has accompanied the growing interest in safety culture surveys.

A few psychometric instruments have been developed to measure organizational patient safety culture, and their strengths and limitations have been reviewed [11-14]. All the existing instruments used Likert scales, and mostly to measured attitudes of individuals towards 4 to 20 dimensions of patient safety culture. The strengths of these tools varied, but only the Safety Attitudes Questionnaire (SAQ) showed links to patient outcomes [11]: favorable scores of the SAQ were associated with fewer medication errors, lower ventilator associated pneumonia, fewer bloodstream infection, and shorter intensive care unit lengths of stay [15,16]. Furthermore, the validity and reliability of the SAQ has been documented in United States (English version) [16,17], United Kingdoms [18], and Norway (Norwegian version)[19].

Although safety activities have been initiated at many hospitals in Taiwan, little is known about the safety culture at these hospitals. Previous local surveys were restricted by methodological limitations, such as unverified survey instruments, small survey scale, and low response rates. To date, no nationwide safety survey has been conducted in Taiwan. The aims of this study were to verify an existing safety culture survey instrument in Chinese and to measure hospital safety culture nationwide.

## Methods

### Adaptation of the SAQ

The Taiwan Joint Commission on Hospital Accreditation (TJCHA) initiated the Taiwan Patient Safety Culture Survey (TPSCS) in 2007. This survey was sponsored and supervised by the Department of Health, Taiwan. A TJCHA taskforce composed of clinical personnel, epidemiologists, and statisticians performed a nationwide survey using the SAQ.

The SAQ was translated to the Chinese version (SAQ-C) from the generic version (Short Form 2006), which contains the following six safety dimensions: teamwork climate, safety climate, job satisfaction, stress recognition, perception of management, and working conditions [19]. Linguistic validation of the translation was performed using the back-translation technique [20]. A pilot validity study was conducted at an academic medical center in Taipei, Taiwan [21]. Analytical results demonstrated that all six dimensions had good reliability. However, the stress recognition dimension was removed from the final version of SAQ-C because its association with safety culture was significantly weaker than that of the other five dimensions. Adding the stress recognition dimension (4 items) did not increase discriminating capability, and removing this dimension did not alter the fit of the whole model [21].

The revised SAQ-C was a single-page questionnaire with 32 core items in five dimensions--teamwork climate, safety climate, job satisfaction, perception of management, and working conditions. The detailed descriptions of the core items in English and Chinese are shown in the Additional file 1. Extra items were added to identify respondents' demographic information (gender, age, job discipline, management role, and working experience in the clinical area) and to determine his or her perception of the following safety behaviors: "I experience good collaboration with nurses in these clinical areas"; "I experience good collaboration with staff physicians in this clinical area"; "I experience good collaboration with pharmacists in this clinical areas"; "Administrators encourage the reporting of medical adverse events in this clinical area", "Managers prioritize safety training programs in this clinical areas", and "Communication breakdowns that leads to delays are common in this clinical area" (reverse question). Responses to all questions were scored on a 5-point Likert scale (1 = disagree strongly, 2 = disagree slightly, 3 = neutral, 4 = agree slightly, 5 = agree strongly).

### Administration of survey

Hospital participation in this survey was voluntary. There were 528 hospitals in Taiwan 2008, including 20 large-scale medical centers providing tertiary care, 77 middle-scale regional hospitals, and 431 small-scale district hospitals. The study taskforce mailed invitations to all hospital superintendents explaining the purpose of the survey. In short, this survey was conducted to elucidate the healthcare workers' attitudes toward the safety culture at their working units and hospitals. Except for hospital identification, no information identifying individuals was recorded; the privacy and confidentiality of all respondents were ensured. Each hospital could receive its own survey results and their comparison to the results of peers. In total, 208 hospitals participated in the survey with signed informed consent. Two hospitals required additional approval by their own institutional review boards.

Because the goal was to survey individuals who had influenced or were influenced by their hospital's culture, inclusion criteria required that respondents work in that particular hospital for a minimum of four weeks. The number of questionnaire sent to each hospital was estimated based on the reported number of employees in the general inpatient units (wards of internal medicine, general surgery, obstetrics and gynecology, and pediatrics) and special units (intensive care units, operating rooms, emergency rooms, pharmacies, laboratories, and radiology departments).

The survey was conducted from May 31 to June 30, 2008. Healthcare workers answered the SAQ-C voluntarily and anonymously. The questionnaires were distributed by hospital coordinators who completed training courses to standardize the administration processes. Each respondent completed his or her own questionnaire at the work unit, and then returned the questionnaire to the hospital coordinators. All questionnaires were sealed in a specially designed envelope and then returned to the TJCHA taskforce by mail before July 30, 2008. The majority of the questionnaires were interpreted by a specially designed optical character-recognition system, but the unclearly written questionnaires were handled manually.

### Data analysis

The internal consistency reliabilities of SAQ-C dimensions were assessed using Cronbach's alpha. Confirmatory factor analysis (CFA) using the two-step structural equation model (AMOS version 5.0 software) was applied to test the extent to which each SAQ-C dimension was explained by items and the extent to which safety culture was explained by the five dimensions. A set of goodness-of-fit indices for the dimension structure model was used, including the comparative fit index (CFI), Tucker-Lewis index (TLI), root mean squared error of approximation (RMSEA), and goodness-to-fit index (GFI).

The scoring system of the SAQ-C was consistent with that in previous studies [18,19]. Each item was scored by converting the 5-point Likert scale to a 100-point scale as follows: 1 = 0, 2 = 25, 3 = 50, 4 = 75, and 5 = 100. Responses to each item within the same dimension were summed and then divided by the number of items in that dimension to create a dimension score in the range of 0-100. If a respondent's mean score was 75 or higher, he or she was reported to hold a positive attitude to a given dimension.

The distributions of the mean percentage of hospital healthcare workers holding positive attitudes to each safety dimension were described and plotted. Using individual as the unit of analysis and considering the clustering effects within the same hospital (by hospital identification number), generalized estimating equation methods with independent working correlation structures [22] were used to explore the association between respondents' safety attitudes (single regression analysis for each SAQ-C dimension and multiple regression analysis for five dimensions together) and perception of their hospital's safety behaviors (good collaboration with nurses, physicians and pharmacists; encouragement of reporting medical adverse events, managers prioritizing safety training, and delays in the delivery of care due to communication breakdowns; agree = 1, disagree = 0). The Wald chi-square test was used to examine the statistical significance between each SAQ-C dimension and safety behavior. All statistical analyses were performed using SPSS version 16 software.

## Results

### Survey responses

In total, 45,242 valid questionnaires were returned from 200 hospitals (8 hospitals withdrew from the study), representing 100% (20/20) medical centers, 74% (57/77) of regional hospitals, and 28% (123/528) of district hospitals of the country. The characteristics of respondents are listed in Table 1. Nearly 65% of respondents were nurses. About 45% of respondents were from medical centers, 36% from regional hospitals, and only 19% from district hospitals. The average response rate was 69.4% (from 52.3% to 86.1%). Nurses had the highest response rate (72.4%), followed by technicians (69.5%), pharmacists (67.2%), administration workers (63.8%), and other staffs (60.8%). Physicians had the lowest response rate of 56.1%.

**Table 1:** Characteristics of safety attitude survey respondents.

Characteristics	Frequency	%
Gender		
Male	5,789	12.8
Female	39,453	87.2
Age groups (years)		
≤20	142	0.3
21-30	22,942	50.7
31-40	14,212	31.4
41-50	5,810	12.8
51-60	1,302	2.9
>60	84	0.2
Unknown	750	1.7
Job discipline		
Physicians	2,656	5.9
Nurses	29,357	64.9
Technicians	4,854	10.7
Pharmacists	2,880	6.4
Administration workers	1,515	3.3
Others	3,980	8.8
Hospitals (number of hospitals)		
Medical centers (20)	20,308	44.9
Regional hospitals (57)	16,337	36.1
District hospitals (123)	8,597	19.0

### Psychometric properties

The Cronbach's alpha was 0.792 for teamwork, 0.816 for safety climate, 0.912 for job satisfaction, 0.874 for perception of hospital management, and 0.785 for working condition. The CFA indicated a good model fit for each dimension and entire safety construct; that is, the GFI, TLI, and CFI were >0.90 and the RMSAE was <0.10 (Table 2).

**Table 2:** Psychometric properties of the SAQ-C by confirmatory factor analysis.

SAQ dimensions	Model Fit Indices			
	
	GFI	TLI	RMSEA	CFI
Teamwork climate	0.99	1.00	0.04	0.99
Safety climate	0.99	0.98	0.04	0.99
Job satisfaction	0.98	0.99	0.06	0.99
Perception of management	0.98	0.97	0.06	0.99
Working conditions	0.99	0.98	0.07	0.99

Overall model	0.98	0.92	0.06	0.99

GFI: goodness-to-fit index; TLI: Tucker-Lewis index; RMSEA: root mean square error of approximation; CFI: comparative fit index

### Survey analysis

**Figure 1:**
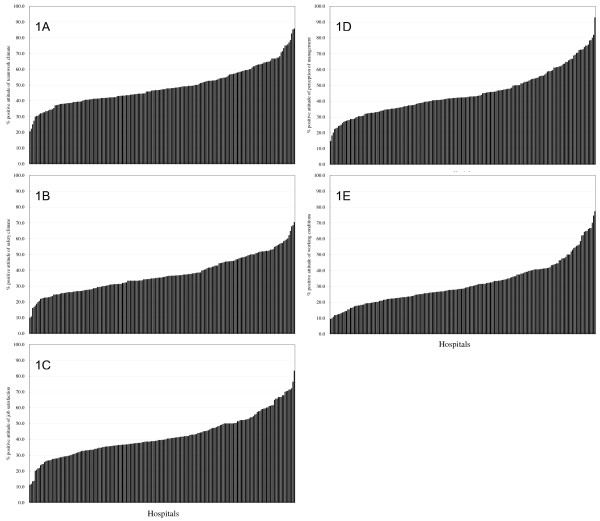
**Figure 1A-1E Distributions of hospital safety culture in Taiwan**. The mean percentage of hospital's employees holding positive attitudes toward teamwork climate (1A), safety climate (1B), job satisfaction (1C), perception of hospital management (1D), and working conditions (1E) of 200 hospitals participated in the Taiwan Patient Safety Culture Survey in 2008.

Table 3 shows the minimum, maximum, and mean percentage of hospital healthcare workers holding positive attitude toward each SAQ-C dimension. On average, 48.9% healthcare workers of 200 hospitals hold positive attitudes toward teamwork climate, followed by perception of management (45.2%), job satisfaction (42.1%), safety climate (37.2%), and working conditions (31.8%). Figure 1 illustrates the distributions and variations in the mean percentage of hospital employees holding positive attitudes toward each safety dimension. The variation within each dimension was the highest in the perception of management (78.2%), followed by job satisfaction (72.2%), working conditions (68.0%), teamwork climate (65.1%), and safety climate (60.4%).

**Table 3:** Perception of hospital safety culture and safety behaviors

	% of hospital employees holding positive attitudes		
	
	Minimum	Maximum	Mean ± SD
SAQ dimensions			
Teamwork	20.6	85.7	48.9 ± 11.8
Safety climate	10.0	70.4	37.2 ± 11.4
Job satisfaction	11.1	83.3	42.1 ± 12.9
Perception of management	14.7	92.9	45.2 ± 13.9
Working conditions	9.3	77.3	31.8 ± 13.5
Safety behaviors Good collaboration with:			
nurses	46.7	98.0	71.1 ± 10.4
physicians	21.2	97.5	60.0 ± 13.4
pharmacists	18.2	93.7	52.4 ± 16.0
Administrators encouraged reporting adverse events	33.3	100.0	77.3 ± 10.5
Managers prioritized safety training	31.3	100.0	70.0 ± 11.6
Service delays due to communication breakdowns were common	0.0	71.4	18.4 ± 8.3

The minimum, maximum, and mean percentage of hospital employees holding positive attitude to each SAQ-C dimension and additional measures of their hospitals' safety efforts.

In terms of the extra measures to hospital safety behaviors, 71.1% of all respondents perceived good collaboration with nurses, higher than that with physicians (60.0%) and pharmacists (52.4%). If nurses were not counted, 68.9% of the respondents perceived good collaboration with nurses, 66.3% felt so with physicians, and 62.9% with pharmacists. Nearly 70.1% of respondents held positive attitudes to their hospitals' efforts in prioritizing safety training, and 77.3% felt positive about administrators' encouragement for reporting adverse events. Only 18.4% of respondents commented that communication breakdowns leading to delays in care delivery were common (Table 3).

Table 4 presents the association between each safety dimension and safety behavior by a multi-level analysis, adjusting for the clustering effects at hospital levels. Compared to those without positive attitudes to teamwork climate, healthcare workers with positive attitudes were more likely to have good collaboration with coworkers; the odds ratio was 1.445 with nurses, 1.498 with physicians, and 1.371 with pharmacists (Wald chi-square test, p < 0.001 of all). The higher the percentage of employees held positive attitudes toward teamwork climate, the more likely their managers encouraged safety events reporting (by 45.2%, p < 0.001) and prioritized safety training (by 36.7%, p < 0.001). Similar associations were also demonstrated between the other SAQ-C dimensions and safety behaviors (Table 4). There were negative but not statistically significant associations between each safety dimension and the "service delay due to communication breakdown was common." The analytic results by multiple regression models are shown in the Additional file 2. The study findings were similar by two different methods.

**Table 4:** Relationships between safety culture measured by the SAQ-C and safety behaviors (positive = 1, negative = 0); comparing the odds ratio (and 95% confidence interval) between those with and without (reference group) positive attitudes to the teamwork climate, safety climate, job satisfaction, perception of management, and working conditions by generalized estimation equation models, adjusting for the clustering effects at hospital levels.

Safety Behaviors	Safety Attitude Questionnaire (Chinese version)				
	
	Teamwork Climate	Safety Climate	Job Satisfaction	Perception of Management	Working Conditions
Good collaboration with					
Nurses	1.445 (1.428, 1.462)	1.416 (1.400, 1.431)	1.438 (1.422, 1.454)	1.469 (1.453, 1.486)	1.453 (1.438, 1.468)
Physicians	1.498 (1.479, 1.517)	1.471 (1.453, 1.489)	1.513 (1.495, 1.532)	1.527 (1.508, 1.546)	1.566 (1.548, 1.585)
Pharmacists	1.371 (1.352, 1.390)	1.422 (1.402, 1.442)	1.445 (1.425, 1.465)	1.489 (1.468, 1.509)	1.527 (1.506, 1.548)
Encouraging safety reporting	1.452 (1.433, 1.471)	1.579 (1.559, 1.599)	1.488 (1.468, 1.507)	1.608 (1.588, 1.628)	1.492 (1.472, 1.512)
Prioritizing safety training	1.367 (1.352, 1.383)	1.406 (1.391, 1.421)	1.357 (1.342, 1.372)	1.465 (1.443, 1.489)	1.447 (1.434, 1.461)
Service delay by communication breakdowns	0.982 (0.971, 0.993)	0.999 (0.987, 1.010)	0.992 (0.981, 1.010)	0.994 (0.983, 1.020)	0.992 (0.984, 1.010)

## Discussion

Although the SAQ has been translated into several languages and has been administrated in the United States, United Kingdoms, and a few European countries [17,19], this is the first time it was translated into Chinese and used in Taiwan. This large study presents the psychometric properties and cross-cultural capabilities of the SAQ scheme. The internal consistency of the SAQ-C is as robust as that of the original English version [16,17] and Norwegian versions [19]. The SAQ-C scheme is also valid based on its good model construct and significant associations with several safety behaviors.

To date, this is the largest safety culture survey carried out at Taiwanese hospitals. The success of the survey is attributable to several factors. First, the SAQ-C was easily answered and there was a high response rate of 69.4%, which is compatible with the international benchmark of 66-72% [16]. Second, the TJCHA fully supported the survey. The TJCHA is the major force advocating patient safety and medical care quality improvement in Taiwan. Hospital leaders were thus more willing to participate in the survey. Third, all respondents were anonymous, and thus they might feel more comfortable to fill out the questionnaire. Fourth, all participating hospitals benefited from the survey as they were provided with feedback information from the survey. Hospitals received not only their own safety culture information, but also the benchmarking data from 200 hospitals, nearly 40% of the total number of hospitals in Taiwan.

Although many patient safety programs have been initiated in Taiwan, these activities mainly focused on technical and engineering solutions to unsafe working procedures and care processes. Nevertheless, many safety efforts would not be implemented and internalized without changes to organizational culture. This study found that safety culture was not sufficiently established at most Taiwanese hospitals. The mean percentages of positive attitudes toward the five safety dimensions were below the international standard (60%). Furthermore, significantly wide variations in all safety dimensions were also noted, which implied that although a few hospitals had already developed a positive safety culture, more hospitals still lagged behind the population mean. The unsatisfactory survey results are warning signals to healthcare authorities, hospital managers, and the public.

Accumulating evidence supports the relationship between mature safety culture and patient safety, and improving a healthcare organization's safety culture is associated with improved patient outcomes [4,23-25]. Therefore, the Joint Commission on the Accreditation of Healthcare Organizations in the United States and the National Patient Safety Association in the United Kingdom suggested hospitals should conduct safety culture surveys for safety improvement on a regular basis. Many U.S. hospitals have utilized valid questionnaires to measure safety attitudes among clinical areas [26,27], and to compare changes in safety attitudes after evidence-based interventions [23,25,28]. However, the combination of safety culture changes and safety initiatives is still rare at Taiwanese hospitals.

There is evidently considerable room for improvement in developing a more mature safety culture than the status quo for most Taiwanese hospitals. This study has shown that the SAQ-C is a valid and easily administrated instrument. For the first step, hospitals can use this tool to measure their employees' safety attitudes on a regular basis. Hospital safety managers can track the trends of culture changes of specific clinical units or the hospital as a whole. Moreover, this study shows that there is strong association between safety culture and healthcare workers' safety behaviors (collaboration, safety training, and adverse events reporting), which are closely linked to patient safety. The regular safety survey at clinical units and hospitals can also be used as leading indicators to reflect to some extent the safety index of the clinical area and the likelihood of adverse events. Hospitals then need to review and take appropriate actions in response to unsatisfactory items based on healthcare workers' collective perceptions of their working areas. For instance, the Comprehensive Unit-based Safety Program was developed and introduced to improve the teamwork climate of intensive care units in Michigan, United States [24]. Accumulating reports have demonstrated the improvement in teamwork can significantly improve patient outcomes and decrease avoidable errors [24,25,28]. More studies are needed in Taiwan to clarify the causal relationship between safety culture changes and clinical outcomes in different culture settings.

The survey results may serve as a reference in formulating national patient safety policies. For instance, less than one-third of the hospital employees felt positive to their working conditions and the level of staffing got the lowest score (32.8%) among all core items. If staff insufficiency was related to hospital's cost containment strategy and the payment scheme of the National Health Insurance, the healthcare authorities could address this safety concern and put it on the agenda of the annual National Patient Safety Forum led by the Department of Health.

Regular hospital safety culture survey after this study has been fully supported by the TJCHA. A SAQ-C website is initiated http://psc.tjcha.org.tw and hospitals can cooperate with the TJCHA to conduct a unit-wide or hospital-wide survey online. The TJCHA taskforce will help in analyzing the survey data and provide feedback to participating hospitals. Continuous efforts have been initiated to minimize the variations in safety culture among hospitals. After the survey, the TJCHA invited hospitals with high SAQ-C scores to share their successful experiences in establishing safety culture with all participating hospitals. Team resource management programs and evidence-based clinical interventions will be developed and introduced by the TJCHA to help hospitals and their healthcare workers in building a safer environment and culture than the status quo, especially for the hospitals with low SAQ-C scores.

## Limitations

Although the study findings were from 45,242 respondents of 200 hospitals, the findings should be interpreted with caution due to the following limitations. First of all, the external validity of the study findings was limited by the study design and participants. We did not adopt a systemic sampling method so that there were more respondents from large and middle-scale hospitals than from small-scale hospitals. Physicians, pharmacists, and other healthcare personnel were under-sampled compared to the nurse population. Nurses had higher perception of good collaboration with their nurse peers than the other occupational groups. The higher the percentage of nurses participating in the survey, the higher the hospital SAQ-C score was the collaboration with nurses but the lower the score with physicians and pharmacists. Moreover, the analytical results were not standardized by their age, sex, jobs, seniorities, working units, and hospital characteristics. More studies are needed to explore the influences of these individual and organizational factors to the safety culture. Third, the reverse question "communication breakdowns that leads to delays are common in this clinical area" was not sensitive enough to detect the communication problems among healthcare workers in Taiwan. We would consider selecting other parameters in the future studies. Fourth, survey results have not been linked to the clinical safety parameters. If additional studies support the correlation between safety culture and clinical safety indicators, the SAQ-C scheme can be developed into a more fundamental and predictive indicator for patient safety than the existing ex-post statistics in regards to medical adverse events.

## Conclusions

The SAQ-C scheme is a valid and reliable instrument for measuring the safety attitudes of healthcare workers in the hospital settings of Taiwan. This large survey provides benchmark data of hospital safety culture and indicates that safety culture is far from established at most Taiwanese hospitals. There is still considerable room for improvement in building a safer culture than the status quo.

## List of abbreviations

SAQ: Safety Attitude Questionnaire; SAQ-C: Safety Attitude Questionnaire in Chinese; TJCHA: Taiwan Joint Commission on Hospital Accreditation; TPSCS: Taiwan Patient Safety Culture Survey; CFA: confirmatory factor analysis; CFI: comparative fit index; TLI: Tucker-Lewis index; RMSEA: root mean squared error of approximation; GFI: goodness-to-fit index.

## Competing interests

The authors declare that they have no competing interests.

## Authors' contributions

WCL extracted and analyzed the data and prepared a draft of the manuscript and contributed to all other aspects of the study. HYW led the TJCHA taskforce and conducted the survey. HHL participated in the design of the study and survey administration. CML, FLC, and HHC collected, extracted and analyzed the survey data. PCW, AF, HCY, and SMH participated in the design of the study. HYW and SMH provided intellectual input into study design and its supervision. All authors have given final approval of the submitted manuscript.

## Pre-publication history

The pre-publication history for this paper can be accessed here:

http://www.biomedcentral.com/1472-6963/10/234/prepub

## Supplementary Material

Additional file 1 Safety Attitude Questionnaire Chinese version.pdf Click here for file

Additional file 2 **Multiple regression models for SAQ-C.pdf**. The two additional files are the Safety Attitude Questionnaire Chinese version and the analytic results of multiple regression models for SAQ-C dimensions. Adobe Acrobat Reader is needed to open and read the file. Traditional Chinese characters are also needed for non-Chinese operating system (Microsoft Windows, Apple Mac, etc.) Click here for file
