# Supplementary material for: Hospital Safety Culture in Taiwan: A Nationwide Survey Using Chinese Version Safety Attitude Questionnaire
Source: BMC Health Serv Res. 2010 Aug 10;10:234. doi: 10.1186/1472-6963-10-234 (PMC2924859; doi:10.1186/1472-6963-10-234)
Supplement: Additional file 1 — Safety Attitude Questionnaire Chinese version.pdf [file 1472-6963-10-234-S1.PDF]

Additional file 1

The mean percentage of positive attitude for each SAQ factor and item of the two-hundred hospitals in Taiwan.

SAQ factors and items	% of positive attitudes	
	Mean	SD
**Teamwork Climate (TW)**		
TW1 Nurse input is well received in this clinical area.<br>本單位護理人員的意見可以充分被接受	52.7	12.0
TW2 In this clinical area, it is difficult to speak up if I perceive a problem with patient care.<br>如果我感覺照顧病人有困難時，在本單位不敢說出來	73.8	8.5
TW3 Disagreements in this clinical area are resolved appropriately (i.e., not who is right, but what is best for the patient).<br>本單位可以妥善地解決臨床上彼此意見的分歧（例如：不是誰對誰錯而是怎麼做對病人最好）。	63.5	9.9
TW4 I have the support I need from other personnel to care for patients.<br>在照護病人時，我可以充分得到所需的支援	64.7	11.3
TW5 It is easy for personnel here to ask questions when there is something that they do not understand.<br>在本單位工作如果有不明瞭的地方很容易可以發問。	78.6	8.7
TW6 The physicians and nurses here work together as a well-coordinated team.<br>在本單位醫師與護理人員是一個同心協力的工作團隊	63.6	13.0
**Safety Climate (SC)**		
SC1 I would feel safe being treated here as a patient.<br>如果我是病人，我認為在這裡會受到十分安全的照護	58.0	13.6
SC2 Medical errors are handled appropriately in this clinical area.<br>在本單位醫療錯誤會被妥善地處理	68.2	11.3
SC3 I know the proper channels to direct questions regarding patient safety in this clinical area.<br>我知道有適當的管道能直接反應與病人安全相關的問題	72.2	10.4
SC4 I received appropriate feedback about my performance.<br>我的工作表現可以得到適當的回饋	46.1	11.7
SC5 In this clinical area, it is difficult to discuss errors.	60.1	11.1

在本單位並不容易討論工作上的錯誤		
SC6 I am encouraged by my colleagues to report any patient safety concerns I may have.<br>同事會鼓勵我去反應任何與病人安全相關的疑慮	59.4	10.5
SC7 The culture in this clinical area makes it easy to learn from the errors of others.<br>本單位的文化會讓人很容易從別人的錯誤中汲取教訓	61.1	8.8
**Job Satisfaction (JS)**		
JS1 I like my job.<br>我非常喜歡我的工作	55.0	12.6
JS2 Working here is like being part of a large family.<br>在本單位工作就像是一個大家庭中的一份子	67.1	11.5
JS3 This is good place to work.<br>本單位是一個很好的工作場所	57.5	13.6
JS4 I am proud to work in this clinical area.<br>我以能夠在這裡工作為榮	57.0	12.5
JS5 Morale in this clinical area is high.<br>本單位的工作士氣很高	48.5	12.8
**Perception of Management (PM)**		
PM1 The unit management supports my daily efforts.<br>本單位的管理部門能充分支持我日常的工作	53.4	12.5
PM2 The hospital management supports my daily efforts.<br>全院性管理部門能充分支持我日常的工作	41.4	13.6
PM3 The unit management doesn't knowingly compromise patient safety.<br>本單位的管理部門不會刻意忽視病人安全	77.2	9.6
PM4 The hospital management doesn't knowingly compromise patient safety.<br>全院性管理部門不會刻意忽視病人安全	74.3	9.5
PM5 The unit management is doing a good job.<br>本單位的管理部門管理做得很好	51.9	12.3
PM6 The hospital management is doing a good job.<br>全院性管理部門管理做得很好	39.4	13.9
PM7 Problem personnel are dealt with constructively by our unit management.<br>本單位管理部門會以建設性的態度來處理發生問題的人員	52.3	13.0
PM8 Problem personnel are dealt with constructively by our	44.6	13.1

hospital management.<br>全院性管理部門會以建設性的態度來處理發生問題的人員		
PM9 I get adequate, timely information about events that might affect my work from unit management.<br>對於可能會影響我工作的事件，在本單位可得到充分且及時的訊息	57.5	12.2
PM10 I get adequate, timely information about events that might affect my work from hospital management.<br>對於可能會影響我工作的事件，可由全院性管理部門得到充分且及時的訊息	45.5	13.2
**Working Conditions (WC)**		
WC1 The levels of staffing in this clinical area are sufficient to handle the number of patients.<br>本單位的臨床人力配置足以處理病人工作量	32.8	14.1
WC2 this hospital does a good job of training new personnel.<br>本單位有做好新進人員訓練工作	57.4	13.3
WC3 All the necessary information for diagnostic and therapeutic decisions is routinely available to me.<br>在做診斷與治療決策時，我都可以獲得所有必需的資訊	52.1	12.5
WC4 Trainees in my discipline are adequately supervised.<br>在我專業領域的受訓人員能得到適當的監督與指導	60.6	12.5
