# Supplementary material for: Hospital Safety Culture in Taiwan: A Nationwide Survey Using Chinese Version Safety Attitude Questionnaire
Source: BMC Health Serv Res. 2010 Aug 10;10:234. doi: 10.1186/1472-6963-10-234 (PMC2924859; doi:10.1186/1472-6963-10-234)
Supplement: Additional file 2 — Multiple regression models for SAQ-C.pdf. The two additional files are the Safety Attitude Questionnaire Chinese version and the analytic results of multiple regression models for SAQ-C dimensions. Adobe Acrobat Reader is needed to open and read the file. Traditional Chinese characters are also needed for non-Chinese operating system (Microsoft Windows, Apple Mac, etc.) [file 1472-6963-10-234-S2.PDF]

Additional file 2

The associations between healthcare workers' safety attitudes and safety behaviors at their hospitals were examined by multiple regression models. Generalized estimation equations (GEE) with independent working correlation structures were used to adjust the potential clustering effects at hospital levels. The five SAQ dimensions (teamwork climate, safety climate, job satisfaction, perception of management, and working conditions) were fitted together as the independent variables, while the six outcome measures (positive=1, otherwise=0) were in binominal distributions with log-link functions in GEE analysis. The results were presented in odds ratios (and 95% confidence interval).

Safety Behaviors	Safety Attitude Questionnaire (Chinese version)				
	Teamwork Climate	Safety Climate	Job Satisfaction	Perception of Management	Working Conditions
Good collaboration with					
Nurses	1.759	1.478	1.781	1.772	2.026
	(1.653, 1.872)	(1.365, 1.600)	(1.648, 1.926)	(1.637, 1.918)	(1.699, 2.392)
Physicians	1.506	1.252	1.543	1.384	1.499
	(1.440, 1.575)	(1.185, 1.323)	(1.460, 1.631)	(1.310, 1.461)	(1.311, 1.702)
Pharmacists	1.149	1.211	1.263	1.334	1.777
	(1.112, 1.188)	(1.159, 1.264)	(1.212, 1.316)	(1.277, 1.393)	(1.683, 1.875)
Encouraging safety reporting	1.363	2.025	1.213	2.217	2.492
	(1.275, 1.457)	(1.828, 2.243)	(1.122, 1.313)	(2.011, 2.444)	(2.473, 2.513)
Prioritizing safety training	1.391	1.871	1.265	2.341	2.447
	(1.312, 1.475)	(1.710, 2.046)	(1.180, 1.356)	(2.140, 2.561)	(2.414, 2.463)
Service delay by communication breakdowns	0.935	0.984	0.994	0.996	0.992
	(0.919, 0.950)	(0.941, 1.011)	(0.982, 1.013)	(0.987, 1.024)	(0.981, 1.009)
